# Pursuit of Optimal Vagal Maneuvers in Stable Supraventricular Tachycardia: A Network Meta-Analysis

**DOI:** 10.5811/westjem.47305

**Published:** 2025-11-26

**Authors:** Surya Sinaga Immanuel, Jesslyn Ellenia Gotama, Yeziel Sayogo, Alvin Sunjaya, Gabriel Tandecxi, Clifford Peter Anthony, Stephanie Aurelia Wirawan, Kevin Wibawa, Leonardo Paskah Suciadi

**Affiliations:** * Atma Jaya Catholic University of Indonesia, School of Medicine and Health Sciences, Jakarta, Indonesia; † Rumah Sakit Hasan Sadikin, Department of Cardiology and Vascular Medicine, Bandung, Indonesia; ‡ Siloam Hospitals Kebon Jeruk, Siloam Heart Institute, Jakarta, Indonesia

## Abstract

**Introduction:**

Vagal maneuvers are first-line therapy for hemodynamically stable supraventricular tachycardia (SVT), yet the relative efficacy of the standard Valsalva Maneuver (SVM), modified Valsalva maneuver (MVM), carotid-sinus massage (CSM), and head-down deep breathing (HDDB) remains uncertain. We undertook a network meta-analysis (NMA) to define the optimal technique and explore age- and sex-related effect modification.

**Methods:**

We searched nine databases from inception to January 2025 for randomized controlled trials involving adults (≥ 18 years of age) with stable SVT treated with at least two of the four maneuvers. Primary outcomes were conversion to sinus rhythm after a single attempt after multiple attempts, and by the end of the trial. Secondary outcomes were the need for rescue intravenous (IV) antiarrhythmic drugs and maneuver-related adverse events (AEs). Bayesian random-effects NMA generated risk ratios (RR) with 95% credible intervals (CrIs); surface under the cumulative ranking curve (SUCRA) quantified hierarchy. We performed consistency, publication bias, and sensitivity analyses, and network meta-regression for mean age and female proportion.

**Results:**

Nineteen trials (n = 2,545) formed a connected network. The MVM was more than doubly effective for single-attempt conversion relative to the SVM (RR 2.71, 95% CrI, 2.26–3.31) and outperformed CSM (RR 6.57, 3.33–14.94) and HDDB (RR 1.30, 0.35–4.66); SUCRA = 88.7%. At the end of the trial, the MVM retained superiority over the SVM (RR 1.25, 1.03–1.56) and ranked the highest success rate (SUCRA = 81.3%). The MVM also reduced IV drug use vs the SVM (RR 0.64, 0.55–0.73) and CSM (RR 0.59, 0.37–0.90). No maneuver differed in multiple-attempt success or AEs. The HDDB technique was ranked highest in safety (SUCRA = 82.4%) but was supported only by a single, small study. Meta-regression showed no age or sex interaction. Inconsistency was minimal; the Egger test suggested small-study effects only for the IV-drug endpoint (*P* = .03).

**Conclusion:**

The MVM provides the greatest likelihood of rapid sinus rhythm restoration and the least need for rescue pharmacotherapy without increasing AEs, supporting its adoption as the default vagal strategy for SVT. Larger, standardized trials are warranted to confirm safety differentials and long-term outcomes.

## INTRODUCTION

Supraventricular tachycardia (SVT) is an arrhythmia originating at or above the atrioventricular node, typically presenting with a narrow QRS complex and a heart rate > 100 beats per minute.^1^ It encompasses three primary subtypes: atrioventricular nodal reentrant tachycardia (AVNRT); atrioventricular reentrant tachycardia (AVRT), and atrial tachycardia. AVNRT is the most frequently observed subtype. Overall, SVT is the second most common tachyarrhythmia.^2^,^3^ Rehron et al reported that approximately 1.26 million individuals in the United States are affected, with women showing higher overall prevalence (70.5 per 100,000 per year) compared to men (44.7 per 100,000).^4^

Although region-wide incidence data remain scarce, clinical reports from South Asia indicate that SVT is a major arrhythmia; for example, hospital series in Pakistan cites SVT as the most frequent arrhythmia, followed by atrial fibrillation and bradyarrhythmias.^5^ Patients often present with palpitations, chest discomfort, dyspnea, lightheadedness, or syncope.^6^ In hemodynamically stable individuals, current European Society of Cardiology and American Heart Association guidelines recommend initiating treatment with a vagal maneuver—often carotid sinus massage (CSM) or the Valsalva maneuver—to restore normal sinus rhythm. If ineffective, intravenous (IV) adenosine and, if needed, other antiarrhythmics (verapamil, diltiazem, or beta-blockers) are administered, while synchronized cardioversion is reserved for hemodynamically unstable cases or pharmacological failure.^7^,^8^ Although typically effective, these medications can cause adverse events (AEs), such as hypotension or bradycardia, making non-pharmacological vagal maneuvers an attractive first-line option.^7^,^9^

Vagal maneuvers can be traced back to 1704 when Antonio Maria Valsalva first described a forced expiration against a closed glottis to modulate cardiac arrhythmias.^10^ Although the standard Valsalva maneuver (SVM) is widely employed, it exhibits inconsistent success rates and can be uncomfortable. Ashraf et al and Günaydın et al reported that the modified Valsalva maneuver (MVM) was more than twice as effective as SVM in restoring sinus rhythm (58% vs 20% and 37.5% vs 17.4%, respectively).^11^,^12^ Similarly, Appelboam et al found that the MVM significantly improved conversion rates to sinus rhythm, from 17% with the SVM to 43% with the modified technique.^13^

The MVM involves completing the strain phase—forced exhalation against resistance, typically into a 10 mL syringe—in a semi-recumbent position, followed by immediate supine repositioning with passive leg raise after the strain. This approach aims to enhance venous return and vagal tone, and the use of a syringe helps standardize the technique.^7^ CSM is often limited by some contraindications, such as recent myocardial infarction, stroke, transient ischemic attack, or significant carotid disease. Further evidence from Huang et al and Abdulhamid et al suggests that the MVM outperforms the standard technique while maintaining a favorable safety profile.^12^,^14^ However, fewer data exist on head-down deep breathing (HDDB), and its effectiveness remains unclear, necessitating further investigation to identify which maneuver optimally balances efficacy and safety.

**Population Health Research Capsule**

What do we already know about this issue?

*Vagal maneuvers are firstline therapy for stable supraventricular tachycardia (SVT), but efficacy is inconsistent.*

What was the research question?

*Among adults with stable SVT, which vagal maneuver maximizes conversion and minimizes intravenous drug use and adverse events?*

What was the major finding of the study?

*The modified Valsalva maneuver provides the greatest likelihood of restoration of rapid sinus rhythm: risk ratio 2.71; 95% CrI, 2.26–3.31; P < .001.*

How does this improve population health?

*Using the modified Valsalva speeds drugfree cardioversion, decreasing emergency department resource use, exposure to IV medications, and potential adverse effects.*

We conducted a network meta-analysis (NMA) to assess the comparative effectiveness of the SVM, MVM, CSM, and HDDB in restoring sinus rhythm, reducing the need for emergent IV antiarrhythmic therapy, and minimizing AEs among adults with stable SVT. A network meta-regression (NMR) further evaluated whether age or sex modifies treatment success. Determining the best vagal strategy could enhance patient outcomes, lessen reliance on pharmacological agents, and lower healthcare costs in managing stable SVT.

## METHODS

### Protocol Registration and Reporting Guidelines

The study was conducted and reported under a prespecified protocol registered in the International Prospective Register of Systematic Reviews (PROSPERO; CRD42025636018). All methods followed the Preferred Reporting Items for Systematic Reviews and Meta-Analyses for Network Meta-Analyses (PRISMA-NMA) guidelines.^15^

### Eligibility Criteria

Eligible studies were randomized controlled trials (RCT) enrolling adults (≥ 18 years of age) diagnosed with stable SVT, including paroxysmal SVT, AVNRT, and AVRT, confirmed by electrocardiogram (ECG). Participants had to receive one or more of the non-pharmacological vagal maneuvers described above as the initial attempt to terminate SVT. Studies were required to compare at least two different vagal maneuvers and to report at least one of the following outcomes: successful conversion of SVT to sinus rhythm after a single attempt, multiple attempts, or by the end of the trial; requirement for emergency IV antiarrhythmic agents; and occurrence of any AEs related to the maneuver. A single attempt was defined as a conversion to sinus rhythm after one application of the maneuver, while multiple attempts referred to the cumulative success after repeated applications of the same maneuver within the acute management period and before any other intervention, such as IV antiarrhythmic therapy or direct current cardioversion. Success by the end of the trial was assessed at the final observation point predefined by each study, typically minutes to hours post-intervention and inclusive of additional therapies. Emergency IV antiarrhythmic drug use was defined as the need for agents such as adenosine, verapamil, or sotalol after failed non-pharmacological maneuvers, and AEs included transient bradycardia, hypotension, syncope, or carotid hypersensitivity directly attributable to the maneuver.

Exclusion criteria encompassed pediatric populations (< 18 years of age), patients with hemodynamic instability requiring immediate electrical cardioversion, structural heart disease, and concomitant arrhythmias. We also excluded studies that reported pharmacological or electrical therapy was used as a first-line or concurrent intervention rather than as rescue therapy following a vagal maneuver. Additionally, studies that did not include at least one of the specified vagal maneuvers as part of a randomized comparison, or employed non-standard or poorly described maneuvers, were not considered. Observational studies, case series, case reports, non-original research, and studies providing insufficient outcome data were likewise excluded.

### Data Sources and Search Strategy

We performed a comprehensive literature search on January 1, 2025, covering nine electronic databases (EuropePMC, ScienceDirect, Google Scholar, PubMed, EBSCOhost, ProQuest, Cochrane Library, Wiley Online Library, and SAGE Journals) from their inception to January 2025. The search used both free-text keywords and Medical Subject Headings terms (eg, “valsalva maneuver,” “vagal maneuver,” “carotid sinus massage,” “head-down deep breathing,” “ice immersion,” “diving reflex,” “retch reflex,” “supraventricular tachycardia,” “paroxysmal supraventricular tachycardia,” “wolff parkinson white,” “randomized controlled trial”), in combination with Boolean operators (“AND,” “OR”). The complete list of keywords is shown in Table S1. In addition, we manually examined reference lists of pertinent articles and reviews to identify any studies potentially missed by the initial database search.

### Study Selection and Data Extraction

All retrieved citations were imported into EndNote X9 (Clarivate Analytics, Philadelphia, PA) for duplicate removal. Three authors (JEG, YS, and SAW) independently screened titles and abstracts, followed by full-text evaluations based on the predetermined eligibility criteria. Discrepancies were resolved through discussion or consultation with senior authors (KW and LPS). Three authors (SSI, AS, and CPA) performed data extraction using a standardized form that captured detailed study information, including the study identifier, publication year, design, database used, study settings, inclusion and exclusion criteria, the definition of SVT, and the maneuver applied (with sample sizes per group). We also recorded baseline participant demographics and clinical characteristics, encompassing mean age (years), percentage of female participants, systolic and diastolic blood pressure in millimeters of mercury (mm Hg), pulse rate (bpm), and the prevalence of type 2 diabetes mellitus, ischemic heart disease, and hypertension. Five principal outcomes were extracted: successful conversion of SVT to sinus rhythm after a single attempt; after multiple attempts; by the end of the trial; the requirement for emergency IV antiarrhythmic agents; and the occurrence of maneuver-related AEs.

### Risk of Bias Assessment

Two authors (SSI and GT) independently evaluated the risk of bias for each included study using the Cochrane risk of bias 2 (RoB 2) tool for RCTs, which encompasses five domains: bias arising from the randomization process; bias due to deviations from intended interventions; bias due to missing outcome data; bias in the measurement of outcomes; and bias in the selection of the reported result. Each domain was rated as “low risk,” “some concerns,” or “high risk.”^16^ Discrepancies were resolved through discussion with the senior authors (KW and LPS).

### Data Synthesis and Statistical Analysis

We conducted all statistical analyses using R v4.4.2 (The R Foundation for Statistical Computing, Vienna, Austria). The primary NMA employed Bayesian random-effects models via the gemtc (Network Meta-Analysis Using Bayesian Methods, R package v1.0-2), BUGSnet (Bayesian inference using Gibbs sampling to conduct NETwork meta-analysis, v1.1.2), and BNMA (Bayesian Network Meta-Analysis using ‘JAGS’, R package v1.6.0) packages.^17^–^19^ We estimated risk ratios (RR) with 95% CrI for all outcomes. Four Markov chain Monte Carlo samplings were each run for 20,000 iterations with a burn-in of 5,000 iterations, and diffuse priors were employed for all parameters. Consistency was appraised via node-splitting techniques.^20^ The surface under the cumulative ranking (SUCRA) curve was generated to rank the interventions for each outcome and visualized using a litmus rank-o-gram.^21^

### Meta-Regression and Sensitivity Analysis

Bayesian covariate NMA was carried out with the gemtc package to explore whether mean age and female percentage (standardized such that one unit equals two standard deviations from the mean) modified the effects of each vagal maneuver on the specified outcomes.^17^ Two additional sensitivity analyses were conducted: one excluding studies deemed to be at high risk of bias; and another employing a frequentist random-effects NMA through the netmeta (NMA using frequentist methods, R package v3.1-1) package to confirm the robustness of the findings.^22^ For outcomes with more than 10 studies, publication bias was evaluated using the Egger regression test with weighted regression and multiplicative dispersion, incorporating the standard error as the predictor. A *P*-value < .05 was considered statistically significant.^23^ Additional sensitivity tables and plots are available from the authors upon request.

## RESULTS

**Figure 1:**
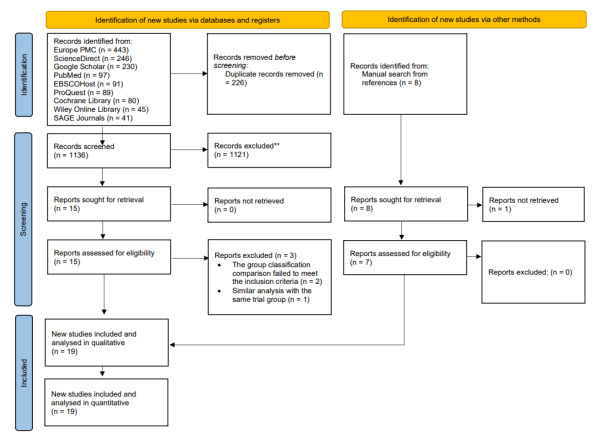
PRISMA flow diagram in a network meta-analysis of vagal maneuvers for stable supraventricular tachycardia. *PRISMA*, Preferred Reporting Items for Systemic reviews and Meta-Analyses.

The database search identified 1,362 records: 443 from EuroPMC; 246 from ScienceDirect; 230 from Google Scholar; 97 from PubMed; 91 from EBSCOhost; 89 from ProQuest; 80 from the Cochrane Library; 45 from Wiley Online Library; and 41 from Sage Journals. After removing 226 duplicate articles, the remaining records were screened, yielding 15 records that were retrieved and assessed for eligibility. An additional manual search of reference lists yielded eight studies from a Chinese database not covered by the international databases, resulting in 23 articles for full-text retrieval. Of these, the full text was unavailable for one study.^24^ Three studies were subsequently excluded, two because of different interventions and one due to duplicate trial data.^25^–^26^ The remaining 19 articles were included in the review.^11^,^13^–^14^,^27^–^43^ The selection process is detailed in Figure 1.

**Figure 2:**
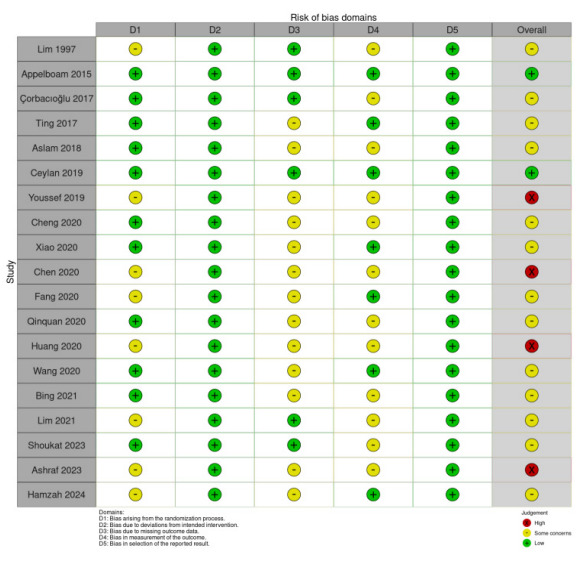
Risk-of-bias summary using the Cochrane risk-of-bias 2 tool for 19 randomized trials of four vagal maneuvers in stable supraventricular tachycardia.

We evaluated all included studies using the Cochrane RoB 2 tool (Figure 2). Among the 19 studies, two were classified as low risk of bias, 13 had some concerns, and four were deemed high risk. Bias arising from the randomization process was identified in seven studies due to insufficient details on their randomization procedures.^27^,^32^,^35^,^36^,^38^,^41^,^43^ Thirteen studies lacked adequate information on participant selection, including pre-intervention exclusions or a detailed schematic of the selection process, leading to potential bias from missing outcome data.^11^,^29^,^30^,^32^,^40^,^43^ Outcome measurement bias was a concern in 11 studies, mainly because AEs were susceptible to subjective interpretation by participants or assessors. In some cases, the assessors were unspecified or not blinded.^11^,^27^,^28^,^30^,^32^,^33^,^35^,^37^,^38^,^40^,^41^ All 19 studies were considered low risk for bias due to deviations from intended interventions and selective outcome reporting. 11,13,27–43

We included 19 RCTs published between 1997–2024 (Table 1). These investigations were conducted in 17 Asian countries and one non-Asian country (Singapore, the United Kingdom, Turkey, China, Egypt, and Pakistan), involving 2,398 patients diagnosed with SVT. Various vagal maneuvers were assessed for SVT termination: the SVM in 18 studies (1,152 patients); the MVM in 17 studies (1,058 patients); CSM in three studies (169 patients), and HDDB in one study (19 patients). The mean age was 51.56 ± 13.73 years, and 54.07% of participants were female. Baseline cardiovascular parameters varied across studies, with a mean systolic blood pressure of 130.70 ± 23.17 mm Hg, a mean diastolic blood pressure of 83.04 ± 15.55 mm Hg, and a mean initial pulse rate of 150.31 ± 48.87 bpm. Comorbidities also varied, including type 2 diabetes mellitus in 21.47% of participants, coronary artery disease in 25.61%, and hypertension in 33.99%. Detailed participant demographics, clinical characteristics, and maneuver protocols are summarized in Table 1.

### Network Meta-Analysis of Single-Attempt Conversion

A total of 15 RCTs (14 two-arm and one multi-arm) involving 1,921 participants evaluated the MVM, CSM, and HDDB in a connected network (Figure S1). Across these trials, 607 successful single-attempt conversion events were reported. The Bayesian network meta-analysis indicated that the MVM had significantly higher single-attempt success than the SVM (RR 2.71, 95% CrI, 2.26–3.31). Both the MVM and SVM, as well as HDDB, also demonstrated significantly greater single-attempt conversion rates compared with CSM (the MVM vs CSM: RR 6.57, 95% CrI 3.33–14.94; HDDB vs CSM: RR 5.06, 95% CrI 1.18–22.26; SVM vs CSM: RR 2.41, 95% CrI 1.24–5.37). Other pairwise comparisons were not statistically significant (Table S2). According to SUCRA values (Table S3), illustrated by the litmus rank-o-gram (Figure S2), the MVM had the highest probability of being the most effective maneuver for single-attempt conversion of SVT (88.72%), followed by HDDB (73.47%), SVM (37.08%), and CSM (0.73%).

We performed sensitivity analyses by excluding studies at high risk of bias, yielding 12 trials (1,693 participants, 517 events), and conducting a frequentist NMA to complement the primary Bayesian approach. In both cases, the network remained connected, and the relative treatment effects were broadly consistent with the Bayesian NMA findings, reaffirming the superiority of the MVM in achieving single-attempt conversion from SVT to sinus rhythm.

### Network Meta-Analysis of Multiple-Attempt Conversion

Eleven RCTs (10 two-arm and one multi-arm) involving 1.752 participants evaluated four maneuvers in a connected network (Figure S1). Across these studies, 213 successful multiple-attempt conversion events were reported. The Bayesian NMA results (Table S2) indicated no statistically significant pairwise differences among the maneuvers. However, the MVM achieved the highest SUCRA value (76.46%), followed by HDDB (60.48%), the SVM (38.99%), and CSM (24.07%) (Table S3, Figure S2). No high-risk-of-bias studies were identified for multiple-attempt conversion; therefore, sensitivity analysis was conducted solely using a frequentist NMA. The network remained connected, and the relative treatment effects were broadly consistent with the Bayesian findings.

### Network Meta-Analysis of End-to-Trial Conversion

Nineteen RCTs (18 two-arm and one multi-arm) involving 2,545 participants evaluated four maneuvers in a connected network (Figure S1) for successful conversion of SVT to sinus rhythm by the end of the trial. Across these studies, 2,051 end-of-study conversion events were reported. The Bayesian NMA showed a statistically significant advantage for the MVM over the SVM (RR 1.25, 95% CrI, 1.03–1.56), whereas other pairwise comparisons were not significant (Table S2). According to SUCRA values (Table S3 and Figure S2), the MVM had the highest probability of being the most effective maneuver (81.30%), followed by HDDB (67.82%), the SVM (36.66%), and CSM (14.22%).

We performed sensitivity analyses by excluding high-risk-of-bias studies—yielding 15 trials (2,079 participants, 1,652 events)—and by conducting a frequentist NMA. In both instances, the network remained connected, and the relative treatment effects were broadly consistent with the Bayesian findings, reaffirming the MVM’s superiority for end-of-trial conversion of SVT.

### Network Meta-Analysis of Intravenous Antiarrhythmic Requirement

Twelve RCTs (all two-arm) involving 1,525 participants compared four maneuvers in a connected network (Figure S1). Across these studies, 1,009 events were reported. The Bayesian NMA showed that the MVM significantly reduced the need for IV antiarrhythmic drugs compared with both the SVM (RR 0.64, 95% CrI, 0.55–0.73) and CSM (RR 0.59, 95% CrI, 0.37–0.90). At the same time, other pairwise contrasts were not statistically significant (Table S2). According to SUCRA values (Table S3 and Figure S2), the MVM had the highest probability of minimizing IV antiarrhythmic requirements (87.07%), followed by HDDB (68.93%), the SVM (28.31%), and CSM (15.69%).

Sensitivity analyses were carried out by excluding high-risk-of-bias studies (yielding nine trials, 1,127 participants, and 762 events) and conducting a frequentist NMA. In both instances, the network remained connected, and the relative treatment effects were broadly consistent with the Bayesian estimates, reinforcing the MVM’s superiority in reducing IV antiarrhythmic use.

### Network Meta-Analysis of Adverse Events

Thirteen RCTs (12 two-arm and one multi-arm study) involving 1,845 participants evaluated four maneuvers in a connected network (Figure S1). Across these trials, 140 events were reported. The Bayesian NMA did not reveal any statistically significant differences among the maneuvers (Table S2), although HDDB achieved the highest SUCRA value (82.41%), followed by CSM (45.52%), the SVM (40.86%), and the MVM (31.20%) (Table S3 and Figure S2). We carried out sensitivity analyses by excluding high-risk-of-bias studies (yielding nine trials, 1,127 participants, and 762 events) and by conducting a frequentist NMA. In both instances, the network remained connected, and the relative treatment effects were broadly consistent with the Bayesian estimates.

### Inconsistency and Publication bias

Node-splitting analyses showed no meaningful disagreement between direct and indirect estimates (all *P* > .05) except for end-of-trial conversion, where CSM vs the SVM (*P* = .03) and the MVM vs the SVM (*P* = .02) indicated significant inconsistency. For IV antiarrhythmic requirements, no comparison contained both direct and indirect data; thus, inconsistency could not be evaluated. Funnel plots and Egger regression revealed no small-study effects for any outcome except IV antiarrhythmic requirement (Egger *P* = .03), indicating small-study effects for this endpoint (Figure S3).

### Network Meta-Regression

A NMR examined whether age or female sex (scaled so that one unit corresponds to a 2 SD increase in the original dataset) influenced the outcomes. All the 95% CrI encompassed zero; hence, age and female did not appear to be statistically significant modifiers of the outcomes.

## DISCUSSION

This NMA establishes the MVM as the most effective vagal strategy for the acute termination of stable SVT. The MVM produced more than double the effectiveness for single-attempt conversion relative to the SVM (RR 2.71, 95% CrI, 2.26–3.31) and retained superiority—albeit with a smaller margin—at the end-of-trial assessment (RR 1.25, 95% CrI 1.03–1.56). The need for rescue IV antiarrhythmic therapy likewise fell by one-third with the MVM compared with the SVM, while CSM and HDDB lagged across all efficacy endpoints. No maneuver differed significantly in multiple-attempt success or adverse-event frequency, underscoring the overall safety of vagal techniques when applied to hemodynamically stable patients.

Our findings align with and extend earlier evidence. Huang et al (2022) and Lan et al (2021) both reported the MVM’s dominance for first-attempt conversion; the present analysis confirms this advantage in a larger evidence base that now includes five post-2021 trials and a fourth maneuver (HDDB).^14^,^44^ The MVM’s physiologic edge likely derives from two complementary components: the forced expiratory strain elevates intrathoracic pressure and vagal efferent tone, while the immediate supine repositioning with leg-raise augments venous return and baroreceptor activation—key determinants of atrioventricular-node refractoriness.^45^

The MVM ranked first for end-of-trial conversion (SUCRA = 81.3%) and was the only maneuver to show a statistically significant benefit over the SVM (RR 1.25, 95% CrI, 1.03–1.56). Credible intervals for every other pairwise comparison crossed unity; thus, no additional maneuver could be declared superior once repeat attempts and pharmacologic rescue were permitted. Consistent with our findings, Huang et al (2022) also reported higher end-of-trial success with the MVM compared with the SVM (RR 2.20, 95% CrI, 1.94=2.50) and CSM (RR 3.62, 95 % CrI 2.04–6.39).^14^,^44^,^46^ The modest inconsistency detected in comparisons involving the SVM probably stems from heterogeneity in strain duration and pressure targets rather than from fundamental differences in therapeutic effect.

The MVM also yielded the most favorable profile for IV drug-sparing. Reduced adenosine or calcium-channel blocker use may translate into shorter emergency department length of stay, fewer hypotensive episodes, and lower cost, reinforcing guideline recommendations that prioritize non-pharmacologic therapy in stable SVT. These findings accord with the meta-analysis by Ahmed et al (2020), which likewise showed higher conversion success and reduced IV antiarrhythmic demand with the MVM vs the SVM.^47^ By contrast, the apparent adverse effects advantage of HDDB—highest SUCRA yet statistically non-significant—derives from a single, small trial and should be viewed as hypothesis-generating. In that study, 89.5% of HDDB recipients and 73.7% of the MVM recipients reported no adverse effects, and no major cardiovascular events occurred in either group.^41^

NMR showed that neither mean age nor female proportion modified treatment effects, supporting the maneuver’s applicability across adult demographic spectra. Nevertheless, future work should explore pediatric and prehospital settings, where ergonomic constraints and patient cooperation differ. By integrating indirect with direct evidence, this analysis offers the most comprehensive quantitative comparison of vagal maneuvers to date. It substantiates current European and American guidelines while providing clinicians with a clear hierarchy that can streamline bedside decision-making: attempt the MVM first; revert to pharmacologic rescue only if conversion fails or contraindications emerge.^7^,^8^

## LIMITATIONS

Several caveats temper these conclusions. First, safety comparisons are underpowered—particularly for HDDB and CSM—owing to sparse event counts and limited study numbers. Second, all included trials focused on immediate or short-term outcomes; data on recurrence, long-term safety, and patient-reported comfort are lacking. Third, the Egger test signaled possible publication bias for the IV-drug endpoint, raising the prospect that small, negative studies remain unpublished. Fourth, dosing and technique variations (eg, strain pressure, duration, leg-raise angle) were incompletely reported, precluding formal subgroup analyses that might refine procedural best practice. Finally, four high-risk-of-bias trials were included because the comparisons provided were essential, and the available data were limited. These trials lacked clear details on randomization, participant selection, and blinding of outcome assessors—particularly for subjective safety outcomes. Their inclusion was necessary to maintain network connectivity and ensure a complete analysis.

## CONCLUSION

The MVM provides the highest likelihood of prompt sinus-rhythm restoration and the lowest reliance on rescue IV therapy among other contemporary vagal techniques, without increasing AEs. When no contraindication exists, it should be adopted as the default first-line maneuver for stable SVT, with the SVM, HDDB, or CSM reserved for situations in which the MVM is impractical or unsuccessful. Larger randomized trials—especially those incorporating standardized procedural protocols and longer follow-up—are needed to confirm safety differentials and to evaluate cost-effectiveness and recurrence prevention.

## Supplementary Information









## Figures and Tables

**Table 1:** Baseline characteristics of 2,545 participants randomized to four vagal maneuvers for stable supraventricular tachycardia.*

Study	Year	Country	Groups	N	Procedure			Age (years)	Female (%)	Initial Pulse (bpm)
					Position	Action/Device	Additional Details			
Lim^27^	Aug 1997	Singapore	SVM	62	Sitting upright	Blowing into a mouthpiece (6-inch tube to sphygmomanometer)	N/A	47.20 ± 18.30	57.40	N/A
			CSM	86	Supine, head tilted to opposite side	Finger pressure with an up–down and postero-medial massaging to compress the carotid sinus	Compresses the carotid sinus between the examiner’s fingers and the cervical vertebrae			N/A
Appelboam (REVERT)^13^	Aug 2015	United Kingdom	SVM	214	Semi-recumbent (45°)	Blowing	Reassessment of cardiac rhythm was done initially by a 3-lead ECG	54.50 ± 16.80	63.00	179.00 ± 29.00
			MVM	214	Initially flat with legs raised at 45° (15.00 s), then semi-recumbent	Blowing	Postural change from flat to semi-recumbent	55.10 ± 16.30	58.00	172.00 ± 29.00
Çorbacıoğlu^28^	May 2017	Turkey	SVM	28	Sitting upright	Deep breath, then blow into a syringe	The patient’s response was assessed by ECG monitoring	49.54 ± 15.62	53.60	178.21 ± 27.34
			MVM	28	Initially sitting, then supine with legs raised at 45°	Deep breath, then blow into a syringe	Sudden switch from sitting to supine with leg elevation	43.97 ± 15.38	64.30	180.35 ± 32.02
Ting^29^	Nov 2017	China	SVM	80	Semi-recumbent	Blowing into a syringe	Maintain the same position for 1 minute	52.00 ± 8.40	30.00	173.00 ± 8.00
			MVM	80	Semi-recumbent	Blowing into a syringe	Change to supine with legs elevated at 45° for 15 seconds (s) and return to semi-recumbent position for 45 seconds	54.00 ± 8.90	28.80	176.00 ± 8.00
Aslam^30^	Dec 2018	Pakistan	SVM	50	Supine	Forcible exhalation against a closed airway (mouth closed, nose pinched)	N/A	40.10 ± 13.10	70.00	N/A
			CSM	50	N/A	Gentle, solid pressure massaging of the right carotid sinus	N/A	38.60 ± 11.80	60.00	N/A
Ceylan^31^	Jun 2019	Turkey	SVM	33	Sitting upright	Deep breath, then push a plunger by blowing into a syringe (connected to a sphygmomanometer)	N/A	57.79 ± 16.27	68.90	167.00 ± 30.99
			MVM	32	Initially sitting, then supine with legs raised at 45°	Deep breath, then push a plunger by blowing into a syringe (connected to a sphygmomanometer)	Sudden change to supine with leg elevation	48.93 ± 19.40	62.20	167.00 ± 31.04
			CSM	33	Supine, head tilted to opposite side	Finger pressure with an upward/downward then postero-medial massaging to compress the carotid sinus	The carotid sinus is located just below the angle of the mandible	62.28 ± 15.49	68.90	165.86 ± 27.89
Youssef^32^	Nov 2019	Egypt	SVM	24	N/A	N/A	N/A	N/A	63.00	N/A
			MVM	16						
Cheng^33^	Feb 2020	China	SVM	33	Semi-recumbent	Blowing into a manometer	Maintain the same position for 1 minute	55.10 ± 2.20	57.58	178.88 ± 1.83
			MVM	33	Semi-recumbent	Blowing into a 10 mL syringe	Change to supine with legs elevated at 45° for 15 seconds and return to semi-recumbent position for 45 seconds	58.00 ± 1.80	69.70	183.94 ± 2.49
Xiao^34^	Apr 2020	China	SVM	20	Semi-recumbent	Blowing into a 10 mL syringe	Maintain the same position for 45 s	53.83 ± 9.61	35.00	N/A
			MVM	20	Semi-recumbent	Blowing into a 10 mL syringe	Lower limb elevation to 45°–90° for 45 s	54.85 ± 9.73	45.00	N/A
Chen^35^	Jun 2020	China	SVM	119	Sitting	Blowing into a 10 mL syringe	N/A	N/A	N/A	N/A
			MVM	119	Supine with legs elevated at 90°	Standard maneuver (blowing implied)	Change to supine with 90° leg elevation			N/A
Fang^36^	Jul 2020	China	SVM	48	Semi-recumbent	Blowing into a syringe	N/A	47.73 ± 9.81	51.10	N/A
			MVM	48	Semi-recumbent	Standard maneuver (blowing implied)	Change to supine with legs elevated at 45–90° for 15 s and return to semi-recumbent position	48.15 ± 8.35	41.70	N/A
Qinquan^37^	Aug 2020	China	SVM	63	Semi-recumbent	Inhale deeply, then blow into a 10 mL syringe	While holding breath	56.00 ± 8.00	50.80	N/A
			MVM	70	Semi-recumbent	Inhale deeply, then blow into a 10 mL syringe	Change to supine with legs elevated at 45° for 3–5 s	55.00 ± 7.00	55.70	N/A
Huang^38^	Nov 2020	China	SVM	34	Semi-recumbent	Blowing into a 10 mL syringe	Maintain the same position for 1 minute	53.20 ± 1.90	53.00	178.67 ± 2.01
			MVM	34	Initially semi-recumbent, then flat with legs raised at 45°	Blowing into a 10 mL syringe	Change to supine with legs elevated at 45° for 15 s, then return to semi-recumbent for 45 s before reassessment	56.00 ± 2.10	59.00	180.83 ± 2.39
Wang^39^	Dec 2020	China	SVM	181	Semi-recumbent	Blowing into a 10 mL syringe	Patient maintains the position for 1 min before reassessment via ECG​	49.29 ± 13.59	59.10	79.18 ± 15.22
			MVM	180	Initially semi-recumbent, then flat with legs raised at 45°	Blowing into a 10 mL syringe	After exhalation, the patient was placed supine with legs raised at 45° for 15 s, then returned to semi-recumbent for 45 s before reassessment via ECG	51.76 ± 12.02	53.30	75.71 ± 18.26
Bing^40^	Mar 2021	China	SVM	31	Semi-recumbent	Blowing into a liquid pressure gauge	Patient maintains the position for 45 s before reassessment via ECG​	52.47 ± 3.30	38.70	N/A
			MVM	32	Initially semi-recumbent, then flat with legs raised at 45°	Blowing into a liquid pressure gauge	After exhalation, the patient was placed supine with legs raised at 45° for 15 s, then returned to semi-recumbent for 30 s before reassessment via ECG	52.63 ± 3.42	41.70	N/A
Lim^41^	Jul 2021	Singapore	HDDB	19	Lie on a flat bed with a head-down tilt of 30–45°	Five deep breaths and breath-holding Repetitions were carried out in one attempt	The patients were instructed to take full, deep breaths and hold them by counting to 10 before exhaling.	50.20 ± 19.00	57.90	174.00 ± 23.60
			MVM	19	Initially semi-recumbent, then flat with legs raised at 45°	Forced expiration through disposable tubing against a digital manometer	Change to supine with legs elevated at 45° for 15 s, then return to semi-recumbent for 45 s before reassessment	54.50 ± 14.30	42.10	173.00 ± 23.50
Shoukat^42^	Apr 2023	Pakistan	SVM	31	Sitting upright	Inhale deeply, then blow into a 10 mL syringe	Patient maintains the position for 1 min before reassessment with cardiac monitor	42.20	54.80	190.00
			MVM	31	Semi-recumbent	Inhale deeply, then blow into a 10 mL syringe	Change to supine with legs elevated at 45° for 15 s, then return to semi-recumbent for 45 s before reassessment with cardiac monitor	45.10	54.80	185.30
Ashraf^11^	May 2023	Pakistan	SVM	50	Semi-recumbent	Blowing into a 20 mL syringe	Patient maintains the position for 1 min before reassessment with ECG at one minute and then at three-minute intervals.	50.66 ± 11.58	56.00	185.70 ± 17.41
			MVM	50	Semi-recumbent	Blowing into a 20 mL syringe	Change to supine with legs elevated at 45° for 15 s, then returns to semi-recumbent for 45 s before reassessment with ECG at one minute and then at three-minute intervals.	46.48 ± 10.81	52.00	184.94 ± 18.20
Hamzah^43^	April 2024	Iraq	SVM	51	Semi-recumbent	Forced expiration into a 10 ml syringe	N/A	49.60 ± 11.88	61.20	N/A
			MVM	52	Initially flat with legs raised at 45° (15.00 s), then semi-recumbent	Blowing	Postural change from flat to semi-recumbent			N/A
Summary¶				2,398				51.56 ± 13.73	54.07	

† Plus-minus values are means ± SD.

¶ Accounting for only the available data.

* Extended baseline variables available on request

*CSM*, carotid sinus massage; *ECG*, electrocardiography; *HDDB*, head down deep breathing; *MVM*, modified Valsalva maneuver; *N/A*, Not Available; *SVM*, standard Valsalva maneuver.
