# Supplementary figures and images for: Pursuit of Optimal Vagal Maneuvers in Stable Supraventricular Tachycardia: A Network Meta-Analysis
Source: West J Emerg Med. 2025 Nov 26;26(6):1667–78. doi: 10.5811/westjem.47305 (PMC12698150; doi:10.5811/westjem.47305)

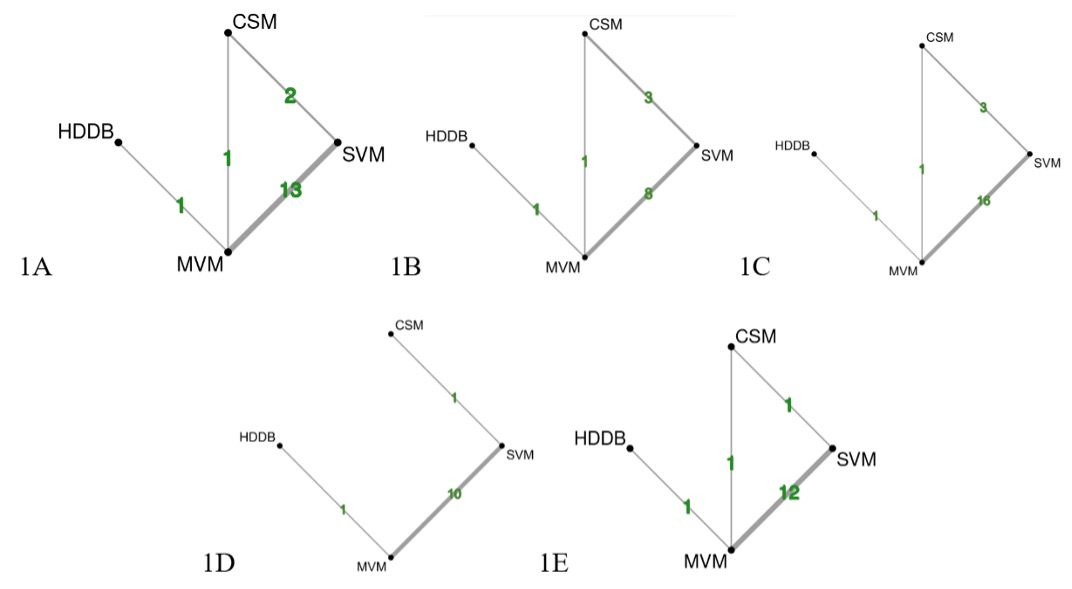

Supplement: Supplementary file 1 [file wjem-26-1667-s001.jpg]

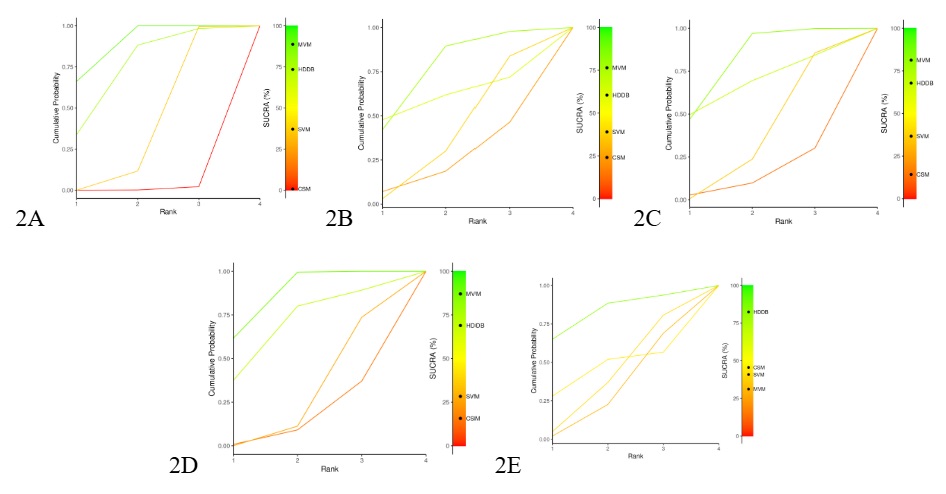

Supplement: Supplementary file 2 [file wjem-26-1667-s002.jpg]

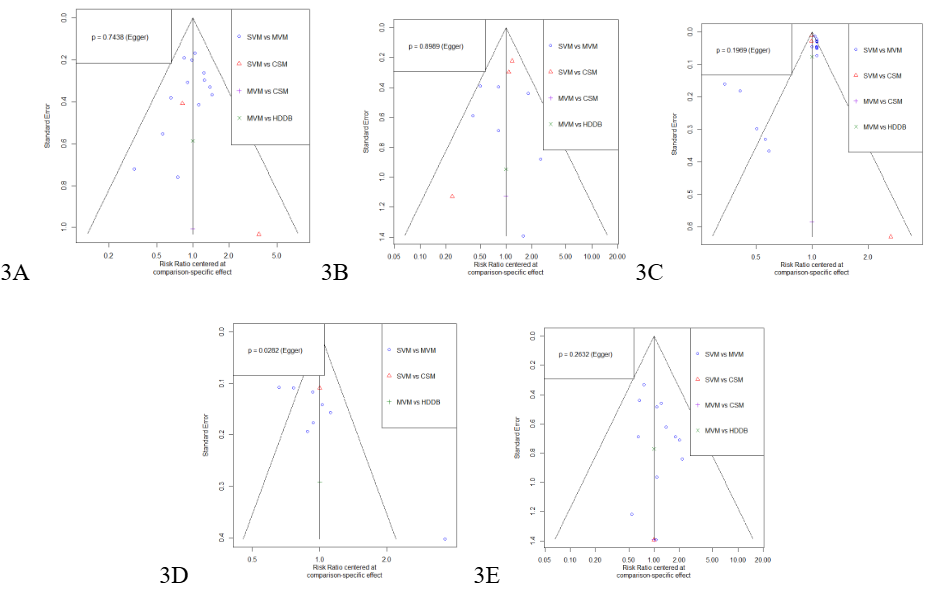

Supplement: Supplementary file 3 [file wjem-26-1667-s003.png]
