# Supplementary material for: Pursuit of Optimal Vagal Maneuvers in Stable Supraventricular Tachycardia: A Network Meta-Analysis
Source: West J Emerg Med. 2025 Nov 26;26(6):1667–78. doi: 10.5811/westjem.47305 (PMC12698150; doi:10.5811/westjem.47305)
Supplement: Supplementary file 4 [file wjem-26-1667-s004.docx]

| **Table S1. Search strategy in a network meta-analysis of vagal manoeuvres for stable SVT.** | | |
| --- | --- | --- |
| **DATABASE** | **KEYWORDS** | **FILTERS** |
| **Pubmed** | ( "Valsalva Maneuver"[Mesh] OR ( valsalva[tiab] AND (maneuver[tiab] OR manoeuvre[tiab] OR maneuvers[tiab] OR manoeuvres[tiab]) ) OR ( modified[tiab] AND valsalva[tiab] AND (maneuver[tiab] OR manoeuvre[tiab]) ) OR "Carotid Sinus Massage"[Mesh] OR (carotid[tiab] AND sinus[tiab] AND massage[tiab]) OR (head[tiab] AND down[tiab] AND deep[tiab] AND breathing[tiab]) OR (breath[tiab] AND holding[tiab]) OR ( vagal[tiab] AND (maneuver[tiab] OR manoeuvre[tiab] OR maneuvers[tiab] OR manoeuvres[tiab]) ) OR (ice[tiab] AND immersion[tiab]) OR (diving[tiab] AND reflex[tiab]) OR ( (gag[tiab] OR retch*[tiab]) AND reflex[tiab] ) ) AND ( "Supraventricular Tachycardia"[Mesh] OR "Tachycardia, Supraventricular"[Mesh] OR (supraventricular[tiab] AND tachycardia[tiab]) OR (paroxysmal[tiab] AND supraventricular[tiab] AND tachycardia[tiab]) OR psvt[tiab] OR avnrt[tiab] OR avrt[tiab] OR (wolff[tiab] AND parkinson[tiab] AND white[tiab]) OR wpw[tiab] OR arrhythmi*[tiab] OR arrhtyhmia[tiab] OR tachyarrhythmi*[tiab] OR tachycardia[tiab] ) AND ( randomized controlled trial[pt] OR controlled clinical trial[pt] OR clinical trial[pt] OR random*[tiab] OR rct[tiab] ) | - |

**Table S2. Bayesian network meta‐analysis results of all five outcomes in a network meta-analysis of vagal manoeuvres for stable SVT.**

|  | **Single-Attempt Conversion** | **Multiple-Attempt Conversion** | **End-of-Trial Conversion** | **Intravenous antiarrhythmic requirement** | **Adverse events** |
| --- | --- | --- | --- | --- | --- |
| CSM vs HDDB | 5.06 (1.18, 22.26) | 1.89 (0.12, 27.34) | 1.52 (0.6, 4.22) | 0.66 (0.28, 1.5) | 0.42 (0.02, 13.12) |
| CSM vs MVM | 6.57 (3.33, 14.94) | 2.00 (0.54, 4.87) | 1.52 (0.93, 2.72) | 0.59 (0.37, 0.9) | 1.15 (0.10, 25.93) |
| CSM vs SVM | 2.14 (1.24, 5.37) | 1.28 (0.43, 2.79) | 1.22 (0.76, 2.08) | 0.92 (0.6, 1.39) | 1.08 (0.09, 24.04) |
| HDDB vs MVM | 0.78 (0.22, 2.7) | 0.98 (0.09, 12.41) | 1.00 (0.44, 2.26) | 0.90 (0.44, 1.82) | 2.81 (0.59, 19.36) |
| HDDB vs SVM | 2.12 (0.58, 7.59) | 1.52 (0.12, 20.11) | 1.25 (0.54, 2.96) | 1.40 (0.68, 2.9) | 2.68 (0.53, 19.33) |
| MVM vs SVM | 2.71 (2.26, 3.31) | 1.56 (0.78, 2.75) | 1.25 (1.03, 1.56) | 1.56 (1.37, 1.83) | 0.94 (0.66, 1.4) |

CSM: Carotid Sinus Massage; HDDB: Head-Down Deep Breathing; MVM: Modified Valsalva Maneuver; SVM: Standard Valsalva Maneuver

*Parentheses values are the risk ratio (95% credible interval)

**Table S3. SUCRA rankings for all five outcomes in a network meta-analysis of vagal manoeuvres for stable SVT.**

|  | **Single-Attempt Conversion** | **Multiple-Attempt Conversion** | **End-of-Trial Conversion** | **Intravenous antiarrhythmic requirement** | **Adverse events** |
| --- | --- | --- | --- | --- | --- |
| CSM | 0.73 | 24.07 | 14.22 | 15.69 | 45.52 |
| HDBB | 73.47 | 60.48 | 67.82 | 68.93 | 82.41 |
| MVM | 88.72 | 76.46 | 81.3 | 87.07 | 31.2 |
| SVM | 37.08 | 38.99 | 36.66 | 28.31 | 40.86 |

CSM: Carotid Sinus Massage; HDDB: Head-Down Deep Breathing; MVM: Modified Valsalva Maneuver; SUCRA: Surface Under the Cumulative RAnking; SVM: Standard Valsalva Maneuver

*Values are in percentage
